# The “Decline and Fall Off”

**Published:** 1895-08

**Authors:** 


### THE "DECLINE AND FALL OFF."

For several years the JOURNAL has called attention to the rapid decadence of the State Medical Association, and has insisted that there must be a cause for it, which should be sought for, and if found, removed; that there must be something essentially wrong in its organization. As we have before pointed out, the membership is hardly ten per cent more now than it was twelve or fourteen years ago, when the writer's observation began; and each year it grows steadily less, notwithstanding the large accessions of new members each meeting. The average of members dropped for non-payment of dues each year exceeds the average of new members admitted. In the twelve years referred to, the record shows that over eight hundred new members have joined; and yet to-day the net gain is less than seventy-five, or six a year since 1882.*

The JOURNAL is pleased to note that the Association has at last been aroused to a sense of the situation, and the necessity of some action to prevent the utter destruction of the body; something inevitable, and only a matter of short time, if the policy of the past is to continue. At the Dallas meeting the President called attention to the matter, and a committee was appointed to report upon it, suggesting the probable causes of want of cohesion, with remedy for same. The following is the report of the committee—Dr. Wooten, chairman—from the advance sheets of the Transactions sent us by Secretary West:

Your committee appointed, upon the recommendation of the President, to suggest means for increasing the interest in and attendance upon the meetings of this Association, beg to report as follows:

In our judgment the first and fundamental requisite for reviving and extending the usefulness and efficiency of the organization, is an intelligent and practical understanding of its objects and reason for existence. The purpose of the State Medical Association is to promote scientific research and investigation in the profession, with a view to the practical application of the great principles of medical science to the daily needs of humanity and the current practice of the physician. To that end its meetings should be devoted to a terse, pertinent and vigorous discussion of recent discoveries, approved experience and well-attested facts

---

*The roll of 1894, after the Austin meeting, shows 354 by actual count, including new members there admitted, and excluding sixty-five dropped subsequent to the meeting. At Dallas (1895) thirty were admitted; but the number dropped—not yet ascertained—will probably exceed the gain.

Page(s) missing

